# Supplementary material for: The Evolution and Characterization of the RNA Interference Pathways in Lophotrochozoa
Source: Genome Biol Evol. 2024 May 7;16(5):evae098. doi: 10.1093/gbe/evae098 (PMC11114477; doi:10.1093/gbe/evae098)
Supplement: evae098_Supplementary_Data [file evae098_supplementary_data.zip › Supplementary_materials.zip!/selected_miRNAs/NoGe_selected_miRNAs/mirdeep_pdf_files/NMRB01000076.1_Notospermus_geniculatus_strain_Ushimado_scaffold76,_whole_genome_shotgun_sequence_2796.pdf]

Provisional ID	: NMRB01000076.1_Notospermus_geniculatus_strain_Ushimado_scaffold76,_whole_genome_shotgun_sequence_2796
Score total	: 106.8
Score for star read(s)	: 3.9
Score for read counts	: 102.1
Score for mfe	: 1.4
Score for randfold	: 0
Score for cons. seed	: -0.6
Total read count	: 212
Mature read count	: 99
Loop read count	: 0
Star read count	: 113

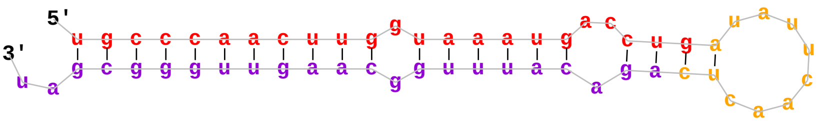

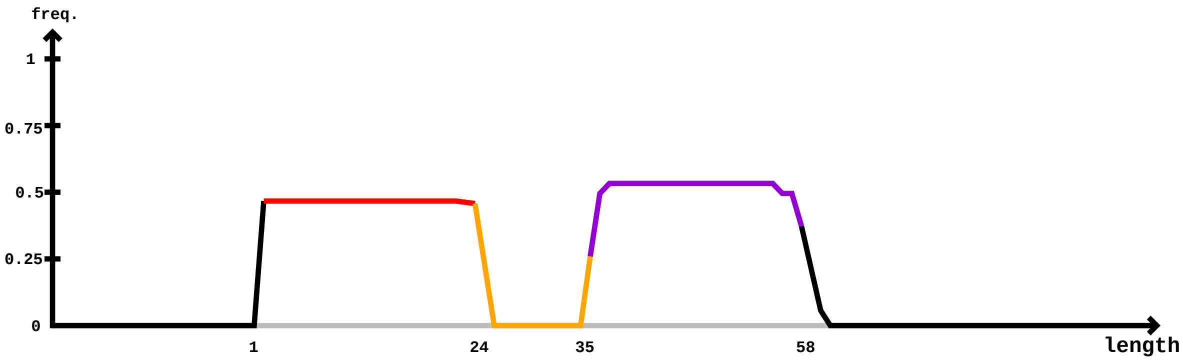

Mature **Star**

```
5'- caauuugauccuuuagugcuugcccaacuugguaaaugaccugauauucaacucagacauuuggcaaguugggcgauuucuuuggguuugaacuugcugaaacacacaaaaga   -3'    obs
    caauuugauccuuuagugcuugcccaacuugguaaaugaccugauauucaacucagacauuuggcaaguugggcgauuucuuuggguuugaacuugcugaaacacacaaaaga          exp
    ((((((((.(((..((...((((((((((((.((((((..((((........)))).)))))).))))))))))))...))..))).))))).))).................    reads    mm    sample
    ....................ugcccaacuugguaaCugacc........................................................................    1        1     Mm3
    ....................ugcccaacuugguaaaugaccuga.....................................................................    1        0     Mm3
    ....................ugcccaacuugguaaCugaccuga.....................................................................    1        1     Mm3
    ......................................................agacauuuggcaaguugggc.......................................    1        0     Mm3
    ......................................................agacauuuggcaaguugggcga.....................................    1        0     Mm3
    ......................................................agacauuuggcaaguugggcgau....................................    2        0     Mm3

    ....................ugcccaacuugguaaCugaccug......................................................................    2        1     Fm3
    ....................ugcccaacuugguaaCugaccuga.....................................................................    7        1     Fm3
    ......................................................agacauuuggcaaguugggcgau....................................    6        0     Fm3
    ......................................................agacauuuggcaaguugggcgaAu...................................    2        1     Fm3
    ......................................................agacauuuggcaaguugggcgauA...................................    2        1     Fm3
    .......................................................gacauuuggcaaguugggc.......................................    6        0     Fm3
    .......................................................gacauuuggcaaguuggUcga.....................................    1        1     Fm3
    .......................................................gacauuuggcaaguugggcga.....................................    12       0     Fm3
    .......................................................gacauuuggcaaguugggcgau....................................    15       0     Fm3
    .......................................................gacauuuggcaaguugggcgauu...................................    3        0     Fm3
    ........................................................acauuuggcaaguugggcga.....................................    5        0     Fm3

    ....................ugcccaacuugguaaaugaccu.......................................................................    1        0     Mm2
    ....................ugcccaacuugguaaaugaccug......................................................................    43       0     Mm2
    ....................ugcccaacuugguaaCugaccug......................................................................    5        1     Mm2
    ....................ugcccaacuugguaaCugaccuga.....................................................................    5        1     Mm2
    ....................ugcccaacuugguaaaugaccugU.....................................................................    8        1     Mm2
    ....................ugcccaacuugguaaaugaccuga.....................................................................    11       0     Mm2
    ......................................................agacauuuggcaaguugggcgU.....................................    1        1     Mm2
    ......................................................agacauuuggcaaguugggcga.....................................    6        0     Mm2
    ......................................................agacauuuggcaaguugggcgau....................................    2        0     Mm2
    ......................................................agacauuuggcaaguugggcgauu...................................    1        0     Mm2
    .......................................................gacauuuggcaaguugggc.......................................    1        0     Mm2
    .......................................................gacauuuggcaaguugggcgau....................................    3        0     Mm2

    ....................ugcccaacuugguaaaugaccug......................................................................    8        0     Mm1
```

```
                    Mature                           Star
caauuugauccuuuagugcuugcccaacuugguaaaugaccugauauucaacucagacauuuggcaaguugggcgauuucuuggguuugaacuugcugaaacacacaaaaga
......................................................agacauuuggcaaguugAgcgau...................................     1      1      Mm1
......................................................agacauuuggcaaguugCgcgau...................................     1      1      Mm1
......................................................agacauuuggcaaguugggcgau...................................     8      0      Mm1
......................................................agacauuuggcaGguugggcgauu..................................     4      1      Mm1
.......................................................gacauuuggcaaguugggcgaAu..................................     9      1      Mm1

....................ugcccaacuugguaaaugaccugU....................................................................     6      1      Fm2
......................................................agacauuuggcaaguugggcgauuu.................................     12     0      Fm2
........................................................acauuuggcaaguugggcgauA..................................     3      1      Fm2

......................................................agacauuuggcaaguugggcgau...................................     5      0      Fm1
```
